# Hepatic transcriptome of the freeze-tolerant Cope’s gray treefrog, *Dryophytes chrysoscelis*: responses to cold acclimation and freezing

**DOI:** 10.1186/s12864-020-6602-4

**Published:** 2020-03-12

**Authors:** M. Clara F. do Amaral, James Frisbie, Raphael J. Crum, David L. Goldstein, Carissa M. Krane

**Affiliations:** 1 Department of Biology, Mount St. Joseph University, 5701 Delhi Ave, Cincinnati, OH 45233 USA; 2 Department of Biological Sciences, Wright State University, 3640 Colonel Glenn Hwy, Dayton, OH 45435 USA; 3 Department of Biology, University of Dayton, 300 College Park Ave, Dayton, OH 45469 USA

**Keywords:** *Dryophytes chrysoscelis*, Freeze tolerance, RNA-Seq, Liver, Cryoprotectant

## Abstract

**Background:**

Cope’s gray treefrog, *Dryophytes chrysoscelis*, withstands the physiological challenges of corporeal freezing, partly by accumulating cryoprotective compounds of hepatic origin, including glycerol, urea, and glucose. We hypothesized that expression of genes related to cryoprotectant mobilization and stress tolerance would be differentially regulated in response to cold. Using high-throughput RNA sequencing (RNA-Seq), a hepatic transcriptome was generated for *D. chrysoscelis*, and gene expression was compared among frogs that were warm-acclimated, cold-acclimated, and frozen.

**Results:**

A total of 159,556 transcripts were generated; 39% showed homology with known transcripts, and 34% of all transcripts were annotated. Gene-level analyses identified 34,936 genes, 85% of which were annotated. Cold acclimation induced differential expression both of genes and non-coding transcripts; freezing induced few additional changes. Transcript-level analysis followed by gene-level aggregation revealed 3582 differentially expressed genes, whereas analysis at the gene level revealed 1324 differentially regulated genes. Approximately 3.6% of differentially expressed sequences were non-coding and of no identifiable homology. Expression of several genes associated with cryoprotectant accumulation was altered during cold acclimation. Of note, glycerol kinase expression decreased with cold exposure, possibly promoting accumulation of glycerol, whereas glucose export was transcriptionally promoted by upregulation of glucose-6-phosphatase and downregulation of genes of various glycolytic enzymes. Several genes related to heat shock protein response, DNA repair, and the ubiquitin proteasome pathway were upregulated in cold and frozen frogs, whereas genes involved in responses to oxidative stress and anoxia, both potential sources of cellular damage during freezing, were downregulated or unchanged.

**Conclusion:**

Our study is the first to report transcriptomic responses to low temperature exposure in a freeze-tolerant vertebrate. The hepatic transcriptome of *Dryophytes chrysoscelis* is responsive to cold and freezing. Transcriptomic regulation of genes related to particular pathways, such as glycerol biosynthesis, were not all regulated in parallel. The physiological demands associated with cold and freezing, as well as the transcriptomic responses observed in this study, are shared with several organisms that face similar ecophysiological challenges, suggesting common regulatory mechanisms. The role of transcriptional regulation relative to other cellular processes, and of non-coding transcripts as elements of those responses, deserve further study.

## Background

Winter in temperate regions poses myriad challenges to vertebrates. While some endotherms endure winter’s low temperatures by elevating metabolic heat production and remaining euthermic, others abandon thermal homeostasis, suppressing metabolism, and tolerating reduced body temperature. For overwintering ectotherms, avoidance of somatic freezing can be achieved by locating protected hibernacula or by supercooling [1]. However, a few temperate-zone ectothermic vertebrates survive winter by tolerating freezing of up to 65% of their body fluids [1, 2]. Somatic freezing generates physiological challenges beyond those encountered by endotherms and non-freezing ectotherms, including osmotic imbalances, compromised oxygen delivery to cells, and the potential for tissue damage from ice. To survive these stresses, freeze-tolerant anurans employ several molecular and physiological strategies [2, 3]. Most freeze-tolerant frogs accumulate cryoprotectants: low molecular weight compounds that reduce ice content, stabilize membranes and proteins, and may serve as metabolic substrates during subzero exposures [2, 4]. Furthermore, although cold acclimation and freezing are associated with metabolic suppression [3], genes related to production and distribution of cryoprotectants are upregulated during these periods [5–7], as are antioxidant enzymes [8] and the cellular stress response [9], partially via upregulation of chaperone proteins [10].

Gray treefrogs, *Dryophytes* (formerly *Hyla*) *chrysoscelis* and *D. versicolor* [11], inhabit eastern North America. In fall, treefrogs shift from arboreal microhabitats to sites below terrestrial leaf litter where, in more northern latitudes, they may experience subzero temperatures and somatic freezing [12, 13]. Upon initiation of freezing, treefrogs mobilize cryoprotective glucose and glycerol derived from liver glycogen catabolism [14–17]. These species also accumulate cryoprotective glycerol and urea during cold acclimation in anticipation of freezing [5, 13, 18–21] a response similar to that observed in freeze-tolerant insects [22] and cold-tolerant fish [23]. In *D. chrysoscelis*, the anticipatory accumulation of cryoprotectant occurs concurrently with changes in kidney function [5] and adjustments in aquaglyceroporin expression and localization [7, 20], which likely facilitate permeation of these solutes into various tissues. The molecular mechanisms regulating cold-acclimation and freeze-tolerance responses in this species remain largely unknown.

Previous studies have used pathway- and gene-specific approaches to investigate molecular regulation of cold and freezing responses in several organisms [5, 6, 24]. The development of high-throughput RNA sequencing (RNA-Seq) permits a transcriptome-wide investigation of responses to a variety of stimuli, in a way that allows for both testing of hypotheses and discovery of genes and responses integrated across multiple pathways (e.g. [25–27]). A transcriptome-level investigation of the hepatic responses of *D. chrysoscelis* to low temperatures may be particularly informative for understanding cold acclimation and freeze tolerance, as the liver is essential in cryoprotectant mobilization during these challenging periods [15]. In this study, it was hypothesized that hepatic pathways related to cryoprotectant mobilization and stress tolerance would be differentially regulated to meet the physiological challenges encountered during fall and winter. To test these hypotheses, a hepatic transcriptome was generated using RNA-Seq and gene expression was compared among frogs that were warm-acclimated (hereafter called “warm frogs”), cold-acclimated (“cold frogs”), and frozen (“frozen frogs”).

## Results

### De novo assembly and annotation

**Fig. 1:**
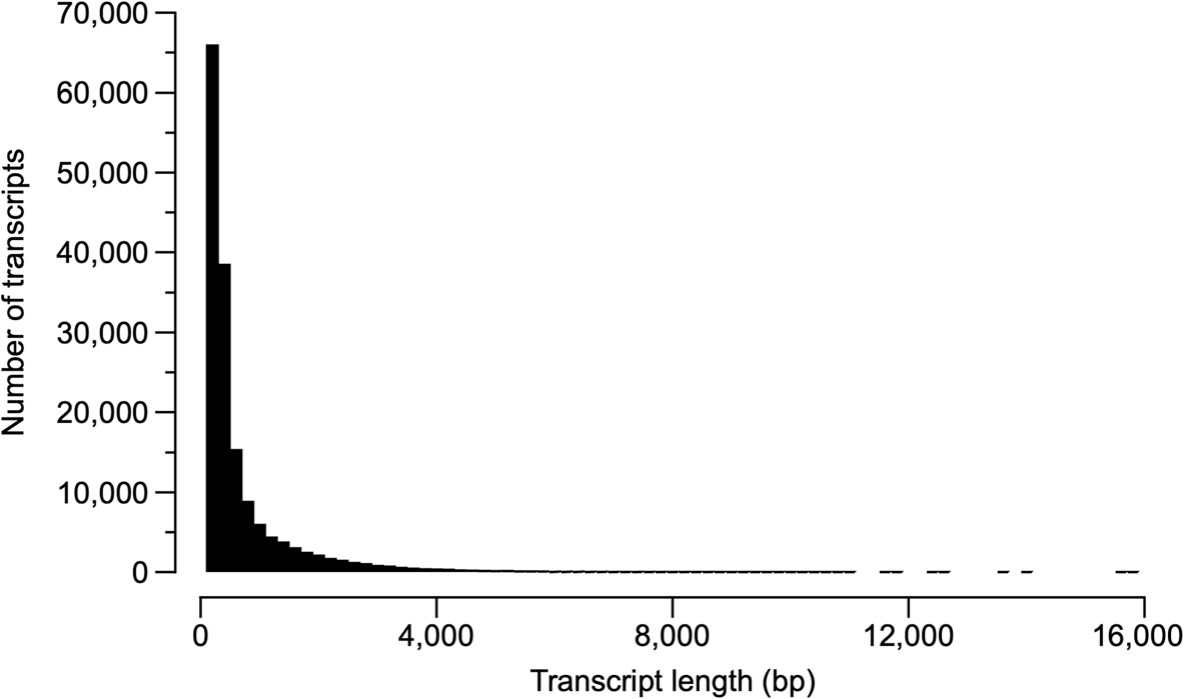
Distribution of transcript length in the transcriptome of *Dryophytes chrysoscelis*

**Fig. 2:**
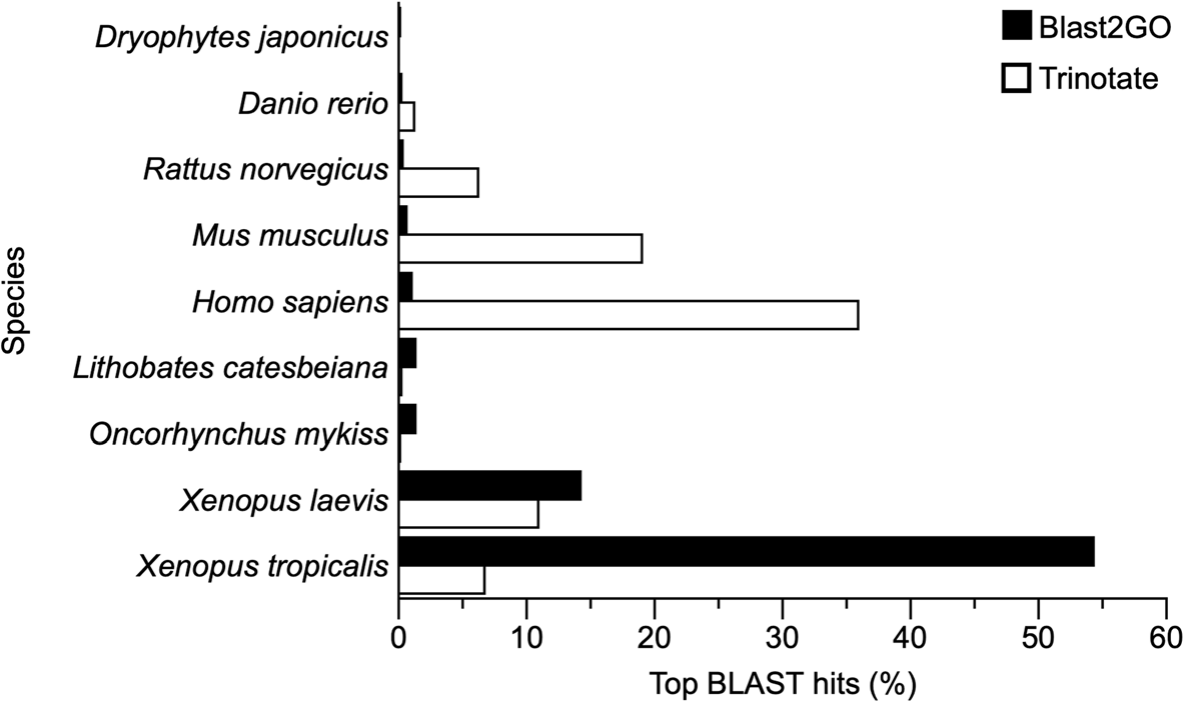
Summary of the species associated with top BLAST hits for the genes in the transcriptome of *Dryophytes chrysoscelis*

A de novo transcriptome was assembled from warm, cold, and frozen frogs (*n* = 4 for all treatments) to increase transcriptional diversity (Table 1). Twelve cDNA libraries were generated from liver tissue of *D. chrysoscelis*. These libraries produced a total of 886 million reads, including 159,556 transcripts, and the assembly had an average read depth of 142. Transcripts were on average 676 bp long, ranging from 201 to almost 16,000 bp (Table 1, Fig. 1). FastQC analyses (FastQC, http://www.bioinformatics.babraham.ac.uk/projects/fastqc/) confirmed per base sequence quality, per sequence quality, and per tile sequence quality throughout the 150 bp sequence length per read for each biological sample, with zero per base N content reported. Following BLASTX (or TBLASTX as appropriate) searches of the Uniprot Swiss-Prot database [28] using Trinotate [29] 52,919 transcripts were identified; using BLAST2GO [30] (BioBam, Spain) an additional BLASTX search of the NCBI vertebrate non-redundant (nr) protein database and of the reference protein sequences (RefSeq) for *Xenopus sp* identified 62,420 transcripts [31]. Combined, the two approaches identified 62,454 transcripts (Table 2). The taxonomic distribution of the BLAST results revealed that, with Trinotate, 18% of transcripts matched *Xenopus sp*. and 36% matched *Homo sapiens* sequences, whereas in Blast2GO, 68 and 1% of the transcripts matched *Xenopus sp*. and *H. sapiens* sequences, respectively (Fig. 2). The Japanese treefrog (*D. japonicus*), a species closely related to *D. chrysoscelis*, was not represented in the Trinotate results (it was absent from the Swiss-Prot database at the time of search) and in Blast2GO accounted for fewer than 1% of transcripts identified.

**Table 1:** Summary of transcriptome data for *Dryophytes chrysoscelis*

Parameters	Value
Total number of reads	886,317,286
Average reads/sample	73,859,774
N50 length of assembly (bp)	1165
Total number of transcripts	159,556
Maximum transcript length	15,730
Average transcript length	676
Average read depth of coverage	142

**Table 2:** Summary of annotation of *Dryophytes chrysoscelis* transcriptome

Parameters	Trinotate	Blast2GO
Transcripts with BLAST hits	52,919	62,420
Transcripts with BLAST hits and annotated	48,865	49,989
Genes identified with BLAST	21,065	34,936
Genes identified with BLAST and annotated	19,705	29,879
Total number of transcripts with a BLAST hit	62,454	
Total number of transcripts with BLAST hit and annotated	53,583	
Total number of genes identified	26,113	
Total number of genes identified and annotated	23,309	

Total numbers were determined by merging the Trinotate and Blast2GO results

A total of 48,865 and 49,989 transcripts were annotated with gene ontology (GO) terms [32] in Trinotate and Blast2GO respectively, and 53,583 transcripts (34% of the total number of transcripts) were annotated when combining the results from both analyses (Table 2). Trinotate identified 21,065 genes, and 19,705 of these were annotated (Table 2). In contrast, Blast2GO identified 34,936 genes, and 29,879 of these were annotated. To maximize coverage, the transcriptome annotated with Trinotate was used, and the genes that were only identified in Blast2GO were added to that transcriptome. Approximately 0.1% of the total transcripts were identified as non-coding functional RNAs upon searching the Rfam database [33] using Blast2GO (Table 3). Of note, the transcriptome of *D. chrysoscelis* included both C/D box and H/ACA box small nucleolar RNAs and 20 different microRNAs: let-7 and miR-7, 10, 15, 16, 22, 23, 24, 27, 29, 30, 101, 122, 142, 144, 145, 194, 214, 221, and 363.

**Table 3:** Non-coding RNA families identified in the transcriptome of *Dryophytes chrysoscelis*

Non-coding RNA	Description	Database hits
cis-reg	cis-regulatory RNAs	3
7SK	7SK small nuclear RNA	1
miRNA	microRNA	20
ribozyme	enzymatic RNA	2
rRNA	ribosomal RNA	4
snoRNA CD-box	antisense box C/D small nucleolar RNA	46
snoRNA HACA-box	box H/ACA small nucleolar RNA	26
snoRNA scaRNA	small Cajal body-specific RNA	3
snRNA splicing	small nuclear RNA, splicing function	2
tRNA	transfer RNA	2

### Differential gene expression

Expression patterns of transcripts and genes in warm (*n* = 4), cold (n = 4) and frozen (n = 4) animals were compared using DESeq2 [34]. We were interested not only in determining which genes showed a significant change (of at least two-fold) in transcriptional output when considering all transcript isoforms, but also which genes had a minimum of one transcript responding to treatment (with at least a two-fold change). We chose this approach as some genes may be identified as differentially expressed without having any of the transcript isoforms show differential expression according to our criteria.

**Fig. 3:**
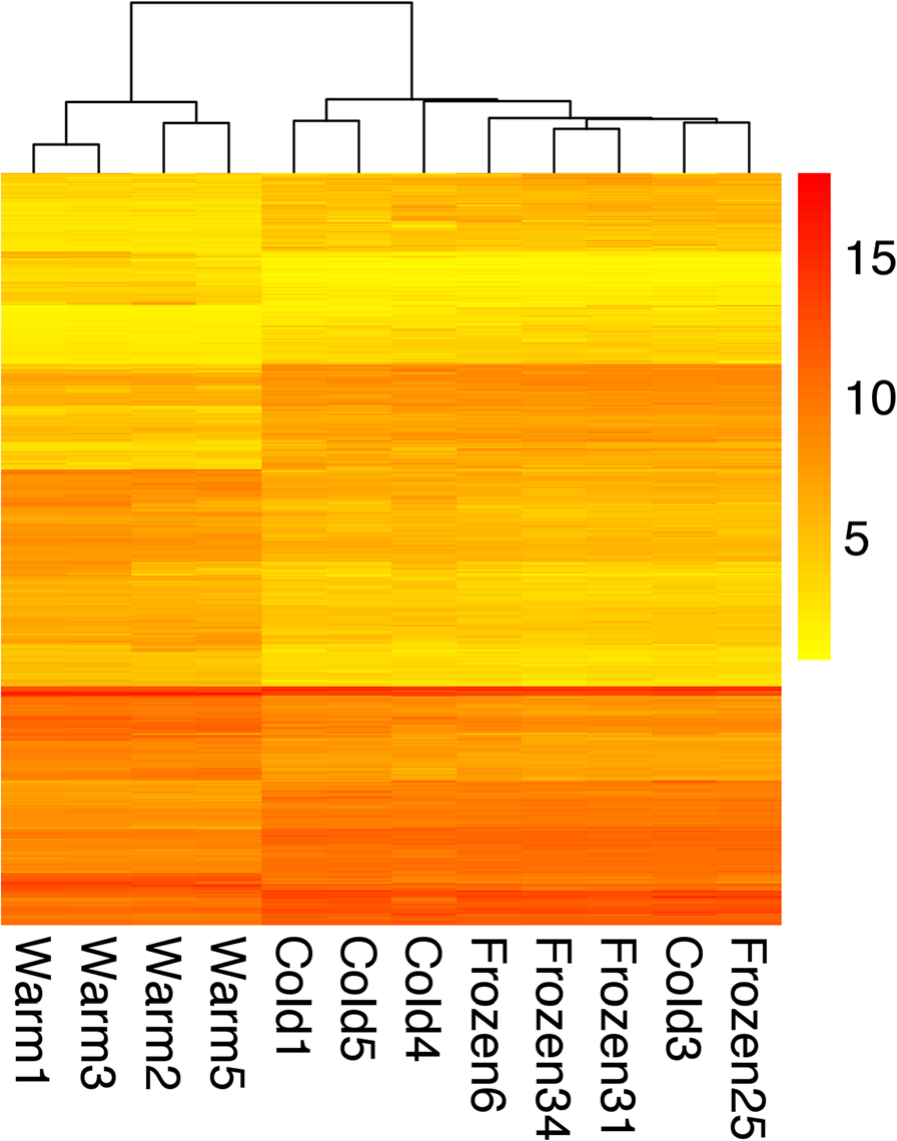
Hierarchical clustering and heatmap of hepatic transcripts differentially expressed among warm, cold, and frozen *Dryophytes chrysoscelis*. The columns represent liver samples and the rows represent differentially regulated transcripts (log2-fold change ≥|2|, FDR < 0.05). Data shown are count data transformed using the *regularized-logarithm transformation* [32]*.* Warm- warm-acclimated (*n* = 4), cold- cold-acclimated (*n* = 4), frozen- frozen frogs (*n* = 4)

Transcript-level analysis followed by gene-level aggregation revealed 3582 genes that were differentially expressed. In contrast, analysis at the gene level revealed 1324 differentially regulated genes. In both transcript and gene-level analyses 1191 genes were differentially expressed. Using transcript-level analysis, cold acclimation resulted in downregulation of 629 genes and upregulation of 1917 genes, whereas freezing resulted in downregulation of 1093 genes and upregulation of 2223 genes, relative to warm-acclimated animals (Fig. 3). Surprisingly, only 18 genes were upregulated in frozen frogs relative to cold animals, and only 7 were significantly downregulated. Gene-level analysis yielded a lower number of differentially expressed genes in all comparisons (Table 4). When contrasting the two analyses, 86–91% of the genes identified as differentially expressed using the gene-level analysis were also differentially expressed in the transcript-level analysis. However, only 20–44% of the genes identified in the transcript-level analysis were shared by both approaches.

**Table 4:** Genes differentially expressed in *Dryophytes chrysoscelis* based on gene-level and transcript-level analyses

		Gene	Transcript	Shared
Cold	decreased	322	629	277
	increased	418	1917	382
Frozen	decreased	596	1093	530
	increased	569	2223	519
Frozen ^a^	decreased	0	7	0
	increased	3	18	3

Genes differentially expressed (log2-fold change ≥|2|, FDR < 0.05) relative to the warm condition except in Frozen^a^, where frozen frogs were compared with cold frogs

As detailed above, a substantial number of sequences were not annotated due to the absence of homology with known genes or transcripts and contained no predicted functional open reading frame. However, a subset of these non-annotated, non-coding sequences (representing ~ 3.6% of the total transcripts) exhibited differential expression in this study when analyzed with all the transcripts (regardless of annotation status). Within this subset, greater than twice the number of sequences were upregulated as were downregulated in liver from cold and frozen frogs relative to warm (Table 5). This regulatory pattern is consistent with that observed with protein coding transcripts (Table 4), suggesting that both coding and non-coding RNAs are subject to thermal state regulation. From these non-annotated transcripts that were differentially expressed, several non-coding RNA families were identified: several C/D box small nucleolar RNAs (SNORDs), H/ACA box small nucleolar RNAs (SNORAs), and two microRNAs, miR-30 and miR-142, were upregulated during cold acclimation and freezing (Table 6).

**Table 5:** Non-coding transcripts differentially expressed in liver tissue from *Dryophytes chrysoscelis*

		transcripts
Cold	decreased	662
	increased	1385
Frozen	decreased	1232
	increased	2507
Frozen ^a^	decreased	6
	increased	8

Transcripts differentially expressed (log2-fold change ≥|2|, FDR < 0.05) relative to the warm condition except in Frozen^a^, where frozen frogs were compared with cold frogs

**Table 6:** Differentially expressed non-coding RNA families

Family	Cold	Frozen	Frozen^a^
snoRNA C/D-box	SNORD12 SNORD14 SNORD22 SNORD29 SNORD30 SNORD31 snoZ17	SNORD12 SNORD14 SNORD22 SNORD29 SNORD30 SNORD31 snoZ17 snoU83B	0
snoRNA H/ACA-box	SNORA5 SNORA28 SNORA68	SNORA5 SNORA28 SNORA66 SNORA68 SNORA79	0
miRNA	miR-30 miR-142	miR-30 miR-142	0

Non-coding RNAs differentially expressed relative to the warm condition except for Frozen^a^, where frozen frogs were compared with cold frogs. All non-coding RNAs listed were upregulated (FDR < 0.05) and none were downregulated

### Functional classification of transcriptome and differentially regulated genes

**Fig. 4:**
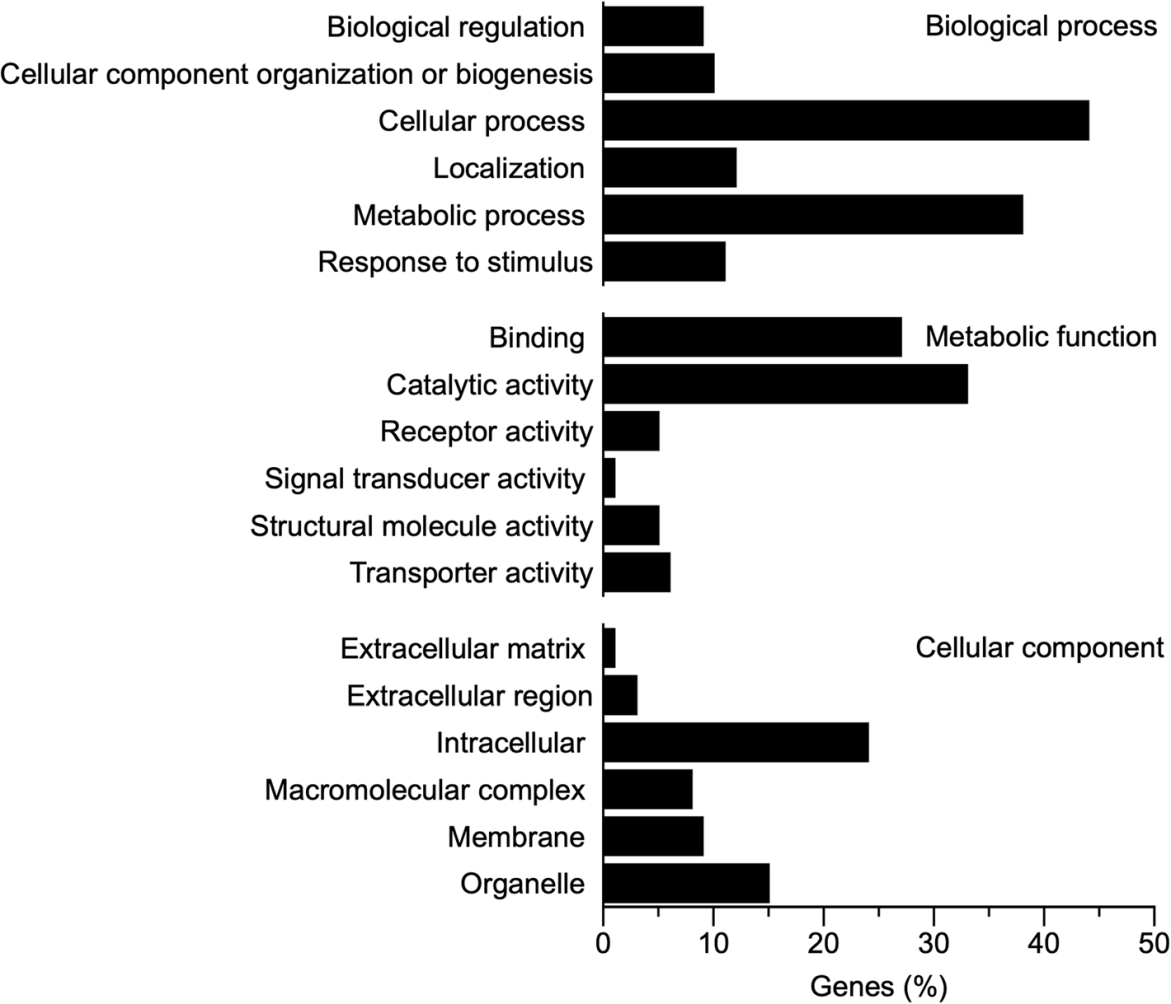
Gene ontology terms describing the transcriptome of *Dryophytes chrysoscelis*. Data shown are percentages of total genes annotated

**Fig. 5:**
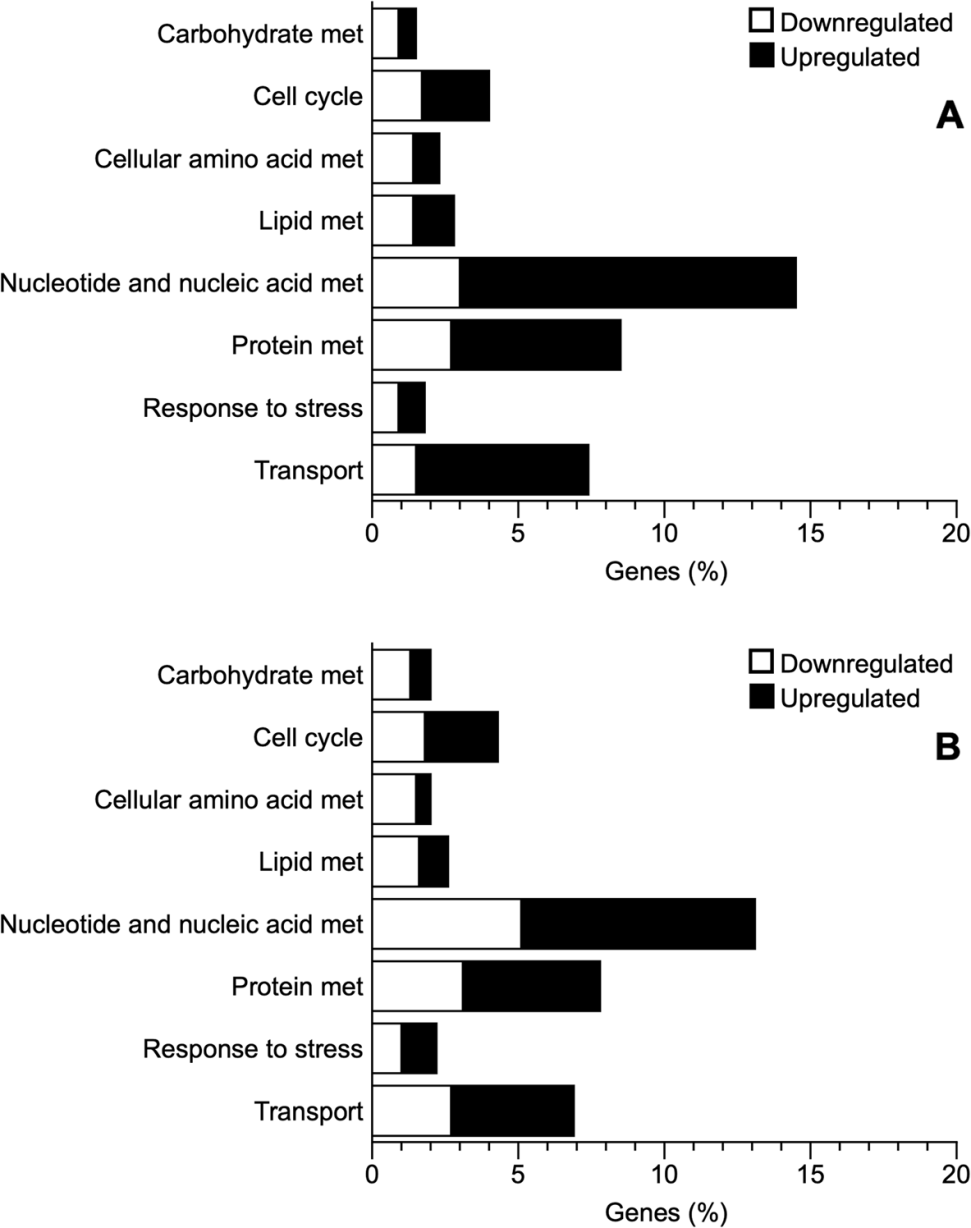
Biological process categories of genes differentially expressed in cold and frozen *Dryophytes chrysoscelis*. Data show the percentage of differentially expressed genes that were up- and downregulated in each treatment. Light bars are downregulated genes and black bars are upregulated genes. **a** cold compared with warm; **b**: frozen compared with warm

**Fig. 6:**
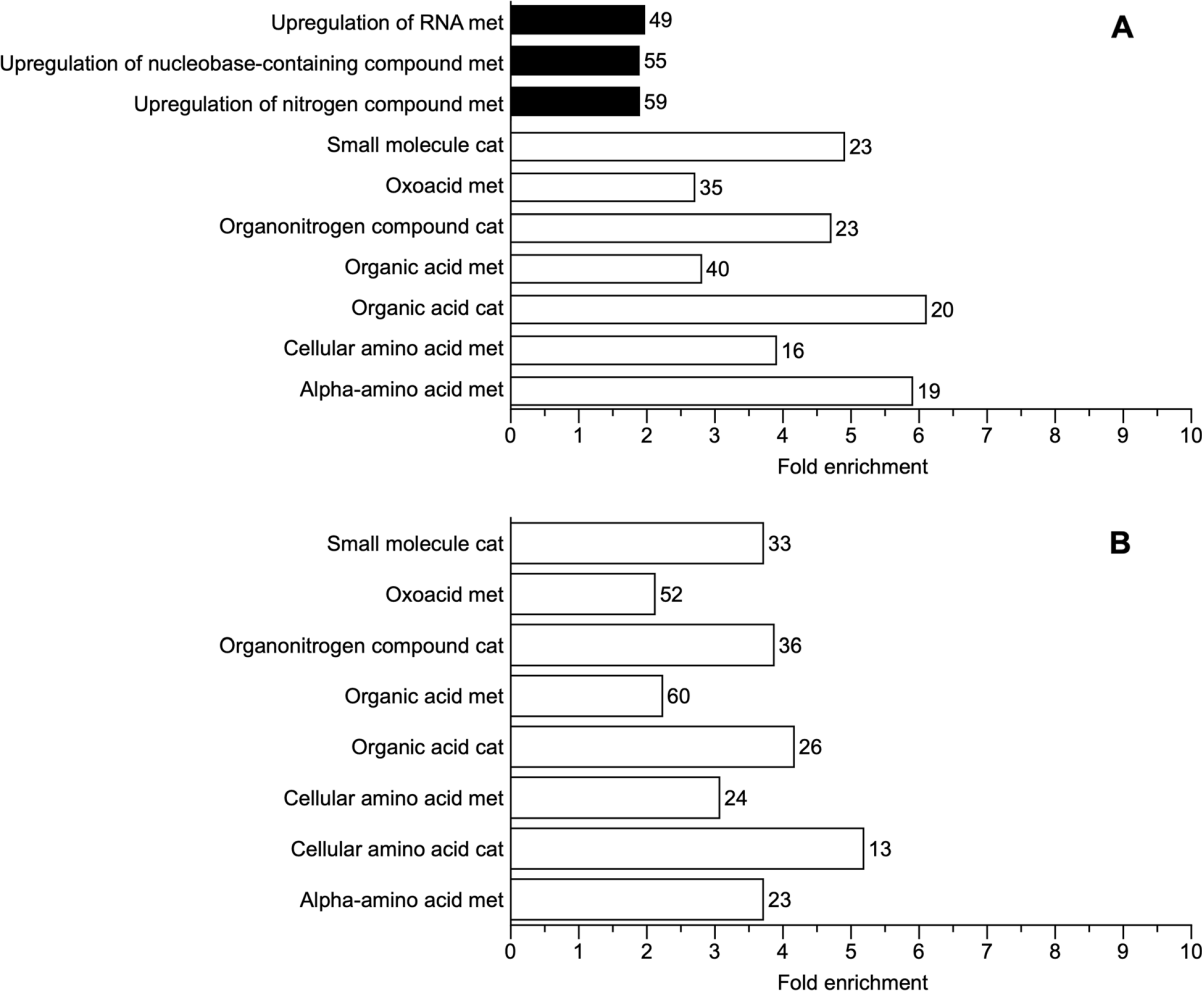
Gene ontology enrichment analysis of biological processes categories in cold and frozen *Dryophytes chrysoscelis*. **a** cold compared with warm; **b** frozen compared with warm. Numbers in front of bars represent the genes in each category. Light bars are enriched categories of downregulated genes and black bars are enriched categories of upregulated genes. A category was considered enriched if the FDR < 0.05

Functional classification of the annotated transcriptome regarding Biological Process categorized 44% of genes as related to cellular processes and 38% related to cellular metabolism (Fig. 4). The most common molecular functions were *Catalytic* (33%) and *Binding* (27%), and approximately 24% of the genes were associated with the cytoplasm and nucleus (*Intracellular*) (Fig. 4). We followed a conservative approach and investigated the genes that were differentially expressed based on both gene- and transcript-level analyses (i.e., genes with at least a two-fold change in transcriptional output and with at least one transcript isoform showing a two-fold change). Cold acclimation and freezing resulted in differential expression of genes related to several GO slim Biological Process (BP) categories including nucleotide and nucleic acid metabolism, lipid metabolism, and stress, among others (Fig. 5; Additional file 1: Table S1). Few genes were differentially expressed between cold and frozen frogs, and thus most of the analysis herein will focus on comparisons with warm animals. Genes related to carbohydrate and lipid metabolism were mostly downregulated during cold acclimation and freezing, relative to warm animals (Fig. 5A and B). Among these were glyceraldehyde-3-phosphate dehydrogenase (*GAPDH*), glycerol kinase (*GLPK*), acyl-CoA dehydrogenase family member 10 (*ACAD10*) and diacylglycerol O-acyltransferase 2 (*DGAT* 2) (Additional file 1: Table S1). However, some genes involved in these same general processes, such as hexokinase (*HK2*) and pyruvate dehydrogenase (*PDK2*), were upregulated (Additional file 1: Table S1). Most genes associated with nucleotide and protein metabolism were upregulated, including proteins associated with ubiquitination, although some genes involved in nucleotide and nucleic acid metabolism, like elongation factor 1-alpha 1 (*EEF1A1*) and signal transducer and activator of transcription 2 (*STAT2*), as well as genes involved in amino acid metabolism, including alanine aminotransferase 2 (*GPT2*) and glutamate dehydrogenase (*GLUD1*), were downregulated in cold and frozen animals (Additional file 1: Table S1). Expression of several transport-related genes was generally higher in cold and frozen than in warm animals, but a few notable exceptions included the solute carrier family 2, facilitated glucose transporter members 8 and 10 (*SLC2A8, SLC2A10*), and sodium/glucose cotransporter 4 (*SLC5A9*) (Additional file 1: Table S1), all of which were downregulated in the cold. Stress-related genes were differentially regulated under cold acclimation (Fig. 5a and b). Specifically, heat shock proteins 90 (*HSP90AA1*) and 70 (*HSP70*) were upregulated in cold frogs, whereas catalase (*CAT*) and caspase 3 (*CASP3*) were downregulated in cold and frozen animals, relative to the warm group (Additional file 1: Table S1). Comparing frozen and cold frogs directly revealed only 3 genes with differential expression: complement component 1Q subcomponent-binding protein, mitochondrial; F-box/LRR-repeat protein 5; and the gene for veficolin-1 (Additional file 1: Table S2). Among genes that were upregulated in cold animals, there was an approximate two-fold significant enrichment (FDR < 0.05) of genes associated with RNA metabolism and with nitrogen compound metabolism. On the other hand, genes that were downregulated in cold frogs were enriched for processes associated with organic acid metabolism and amino acid metabolism, a pattern also detected in frozen frogs, relative to warm animals (Fig. 6a and b).

## Discussion

Temperature is one of the most pervasive factors influencing the physiology of ectotherms. Cope’s gray treefrog, *Dryophytes chrysoscelis*, undergoes molecular, cellular, and physiological adjustments during cold exposure that ensure functional integrity during corporeal freezing [3]. In addition to the metabolic suppression induced by low temperatures, physiological challenges associated with internal ice formation include dehydration and anoxia, along with possibilities of oxidative stress and physical damage from ice [2, 3]. The liver plays a paramount role in anuran cryoprotectant mobilization, a process essential for freezing survival [2, 3]. The molecular processes involved in hepatic responses to low temperature and freezing are complex and not completely understood. The present study investigated responses of the hepatic transcriptome to cold acclimation and freezing in *Dryophytes chrysoscelis*, finding that several cellular and metabolic processes are transcriptionally responsive to thermal changes, potentially promoting cryoprotectant mobilization, stress responses, and transcriptional repression. The environmental and organismal changes that occur during the shift from warm to cold condition— low temperature, anoxia, dehydration, reduced photophase, and aphagia— may have promoted these responses [2, 3].

### The hepatic transcriptome of *D. chrysoscelis*

The RNA-Seq analysis undertaken in this study relied on a de novo transcriptome assembly, generating a high number of transcripts and an N50 on par with those in other RNA-Seq studies that involved a de novo assembly in amphibians (anurans or urodeles) [27, 35, 36]. Two different approaches were used for transcriptome annotation: one approach used Trinotate, which relies on Swiss-Prot as the main database for BLASTX searches; the other relied on Blast2GO to BLASTX sequences on NCBI’s nr protein database, as well as *Xenopus sp.* RefSeq. Blast2GO identified more transcripts than Trinotate, and the taxonomic distribution of the BLAST hits differed between the two approaches, reflecting the inherent bias of species’ representation in the Swiss-Prot database (which contains more than 20,200 *H. sapiens* sequences and approximately 6300 anuran sequences, but the latter representing few genera other than *Xenopus*).

Approximately 34% of transcripts in the present assembly were annotated, within the range of values (32–57%) reported in similar studies [27, 35–37]. In *D. chrysoscelis*, the most common Metabolic Function category in the transcriptome was *Catalytic*, as also was observed in the transcriptome of the green-striped burrowing frog (*Cyclorana alboguttata*) [26]. In contrast, in the crab-eating frog (*Fejervarya cancrivora*) and the green odorous frog (*Odorrana margaretae*), *Binding* was the most common metabolic function [27, 35]. As in other studies reporting amphibian transcriptomes [27, 35, 36], *Cellular* and *Metabolic Processes* were the most abundant Biological Process categories in the hepatic transcriptome of *D. chrysoscelis*. The present study focused on the hepatic transcriptome, whereas other studies have analyzed and reported a merged gene ontology classification of multiple tissues [27, 35, 36]; despite the distinct approaches and evolutionary histories of the species analyzed, the overall composition of the transcriptomes of *D. chrysoscelis* and these anurans is similar.

In addition to protein-coding genes, we also identified transcripts representing several categories of non-coding RNAs. These included several small nucleolar RNAs (snoRNA); 20 different microRNAs, several of which (miR-16, 24, 30, 145, 145, 214, and 363) were also identified in other freeze tolerant amphibians [38, 39] and a number of putative long non-coding RNAs.

### Differential gene expression in liver of *D. chrysoscelis*

Analysis of differential transcript and gene expression was conducted on the 34% of the transcriptome that was annotated. Our criterion for differential gene expression was fairly conservative, as we did not only want to detect genes that had at least one transcript showing differential expression, but that also had an overall change in transcriptional output [40]. In response to cold acclimation and freezing, 5.1% of the annotated genes (5.6% under the gene-level analysis and 15.3% under the transcript-level analysis) were differentially expressed in *D. chrysoscelis.* Collapsing alternative transcripts into a gene-based analysis allowed for a more direct comparison with other published studies. However, in doing so, the gene-level analysis underrepresents the complexity of acclimation-state dependent differential gene expression. Transcript-level analyses revealed 2–4 fold the number of differentially expressed sequences relative to those identified through the gene level analysis (Table 4). The identification of alternative transcripts, and therefore, potentially different protein isoforms that are differentially expressed suggests a role for alternative mRNA processing as a component of acclimation-state dependent gene regulation.

Most differentially expressed genes were found when comparing either cold or frozen frogs to warm animals. Interestingly, 56% of differentially expressed genes (75% of differentially expressed transcripts) showed an increase in expression in cold- vs. warm-acclimated animals. The number of differentially expressed genes and transcripts was further increased in frozen animals relative to warm-acclimated treefrogs, though the number of up- and downregulated genes was similar; in contrast the number of upregulated transcripts was double that of those downregulated, relative to warm-acclimated animals. Only a few genes were differentially expressed between cold and frozen frogs, regardless of the analysis used. These results suggest that physiological adjustments required to survive freezing are initiated during cold acclimation, but that, under conditions of low temperature and metabolic suppression at the time of ice formation, further genomic response is limited. Although a longer duration of freezing might have induced additional transcriptomic adjustments, transcription and translation of new proteins may proceed slowly enough at these low temperatures that other, less energetic mechanisms, such as epigenetic modifications, signal transduction, and actions of microRNAs, predominate [39]. Due to the few differences in gene expression between cold and frozen frogs, the remaining discussion focuses mostly on the comparisons of cold and frozen frogs vs. warm animals.

### Differential expression of genes involved in cryoprotectant mobilization

Glycerol is one of the major cryoprotectants in *D. chrysoscelis*, and this metabolite can be accumulated in the fall [5], and upon initiation of freezing [6], likely as a result of hepatic glycogen catabolism [15, 17]. In this study we found evidence of transcriptional regulation of glycerol mobilization pathways, as enzymes associated with carbohydrate metabolism, and glycerol metabolism in particular, showed differential expression, a pattern found at the transcript and gene-level analyses. Counter to our expectations, that response did not entail enhanced expression of genes promoting glycerol synthesis. Those enzymes, including glycerol-3-phosphate dehydrogenase and glycerol-3 phosphatase, did not show differential gene expression under cold or frozen conditions. Further, in the pathway for glycerol biosynthesis, transcript levels of the key enzyme glyceraldehyde-3-phosphate dehydrogenase (*GAPDH*) were diminished in cold and frozen animals. Downregulation of this gene, along with decreased gene expression of glutamate dehydrogenase (*GLUD1*) and alanine aminotransferase (*GPT2*), suggests a reduced role for glyceroneogenesis in glycerol accumulation in cold and frozen frogs [23]. At the same time, expression of glycerol kinase (*GK*), an enzyme essential for the phosphorylation and metabolism of glycerol in liver, was decreased in cold and frozen animals. Downregulation of *GK* transcription likely decreases glycerol metabolism and thereby contributes to its accumulation for cryoprotective purposes. Curiously, expression of another member of the glycerol kinase family, glycerol kinase 5, was elevated in cold and frozen animals. The function of this kinase in treefrogs is currently unknown but in mice the enzyme is restricted to the skin and has some glycerol kinase activity [41]. A decrease in expression of genes associated with amino acid metabolism is further supported by the GO enrichment analysis which shows downregulated genes being enriched in amino acid metabolism processes. Collectively, these findings suggest that glycerol accumulation during cold acclimation and freezing does not involve transcriptional upregulation of hepatic glycerogenesis, but rather a combination of other regulatory mechanisms, potentially including diminished glycerol metabolism, provision of glycerol from non-hepatic sources like fat bodies, or regulation of transport and distribution of the metabolite.

Transmembrane transport of glycerol occurs largely via aquaglyceroporins, a subclass of the aquaporin protein family that confers permeability to glycerol and water [5, 20]. In previous studies of *D. chrysoscelis*, cold acclimation induced increases in hepatic transcript levels of HC-1 (a water-permeable aquaporin) and HC-3 (an aquaglyceroporin permeable to water and glycerol), and these increases accompanied a cold-induced increase of glycerol in plasma [5]. Hepatic protein levels of HC-9, another glycerol-permeable aquaglyceroporin, also increased during freezing in * D. chrysoscelis* [42], without an associated change in transcript levels. We did not detect changes in transcript levels of either aquaglyceroporin (*HC-3* or *HC-9*) in the current study. The contribution of aquaglyceroporins to glycerol mobilization (export from hepatocytes, uptake in other tissues) may occur via post-transcriptional mechanisms, such as trafficking of the proteins between cytoplasm and plasma membrane.

In treefrogs, mobilization of cryoprotective glucose takes place when freezing begins [14, 15], a process likely triggered by an enzymatic cascade that results in glycogen breakdown and glucose accumulation [3]. Glycogen breakdown in *D. chrysoscelis* is transcriptionally promoted by an increase in expression of phosphorylase kinase b (*PHKA2, PHKG2*) in frozen frogs, and of the β catalytic subunit of protein kinase A (*PRKACB*), which may further potentiate glycogenolysis and subsequent accumulation of glucose and glycerol [4, 6]. In *D. chrysoscelis*, glucose export may also be promoted transcriptionally through the upregulation of glucose-6-phosphatase (*G6PC*), which possibly increases glucose available for hepatic export [4]. Glucose accumulation and export is potentially further augmented by the increased expression of pyruvate dehydrogenase kinase 2 (*PDK2*), which regulates the pyruvate dehydrogenase complex thus inhibiting the oxidative decarboxylation of pyruvate to acetyl-CoA, as well as downregulation of glycolytic enzyme genes like *PFKM* and *GAPDH*. The potential downregulation of glycolysis at the level of the hepatic transcriptome reinforces the enzymatic suppression of this process, a response previously reported in other anurans [4]. Curiously, expression of glucokinase regulatory protein gene (*GCKR*) decreased in cold and frozen frogs, and if reflected at the protein level, would favor glycolysis, a pattern that may be reinforced by the increase in hexokinase (*HK2*) also detected in cold and frozen frogs.

Facilitative glucose transporters (GLUTs), members of the *SLC2A* gene family, have an important role in ensuring the export and import of glucose in various tissues and possibly contribute to the flux of hepatic cryoprotective glucose during freezing. Transcript levels of *GLUT 8* and *GLUT 10* decreased during freezing in *D. chrysocelis*; however, the role of these transporters in anuran glucose homeostasis is unknown. In contrast, *GLUT2*, a major hepatic glucose transporter in vertebrates, was not responsive to low temperature exposure or freezing in this study, although protein and transcript levels of *GLUT2* increase during freezing in *R. sylvatica* [43]. The absence of a freezing response by *GLUT2* transcripts in *D. chrysoscelis* may be in part responsible for the low hepatic export of glucose we detected in a previous study [15]. In addition, *SGLT4*, a Na^+^-dependent glucose transporter (member of the *SLC5A* gene family) was downregulated in frozen animals, compared with the warm condition, suggesting further reduction of hepatic import of glucose during a period when glucose export is being maximized.

Overall, our results suggest a complex, multi-level control of expression of transcripts governing cryoprotectant accumulation, involving regulation of genes involved in cryoprotectant synthesis, metabolism, and transport. The temperature-sensitivity of these transcriptional processes, and also of those involved in synthesizing and processing the proteins that derive from those transcripts, ultimately determines the seasonal pattern of cryoprotectant accumulation. The timing of regulated changes in gene expression may well reflect the ability of various biochemical processes—transcription, translation, transport, enzyme-mediated metabolism, and others—to respond adequately to physiological demands at temperatures near or below freezing.

### Differential expression of genes involved in stress response

Low temperature exposure and freezing result in drastic changes in the physiology of organisms, triggering a series of reactions common to the cellular stress response [9]. In this study, several pathways related to cellular damage showed differential regulation under cold and freezing exposure. Heat shock proteins 70 (*HSP70*), 90 (*HSP90AA1*), 105 (*HSPH1*), as well as the 78 kDa glucose-regulated protein *HSPA5*, were upregulated in cold and frozen frogs. Chaperone proteins function in translocation, folding and assembly, and targeting of misfolded proteins for degradation [9]. Although the HSP response has not been previously investigated during freezing in freeze-tolerant anurans, physiological challenges that occur during freezing, including low temperature, anoxia, and dehydration, do trigger upregulation of HSPs in several species of ectotherms [44, 45], including other freeze-tolerant frogs [10].

The cessation of circulation and ventilation during freezing eventually renders the hepatic tissue anoxic [3]. However, in this study, levels of hypoxia-inducible factor 1 subunit alpha (*HIF1A*), which plays an essential role in hypoxia response [46] and is upregulated in heart of frozen *R. sylvatica* [3], did not change during freezing (data not shown), perhaps reflecting tissue-specific responses.

Low temperature exposure and freezing are likely to trigger oxidative stress and formation of reactive oxygen species, not only due to metabolic suppression, but also during oscillations in tissue oxygen levels resulting from freezing and thawing [3]. Surprisingly, we detected decreases in expression of genes encoding catalase (*CAT*), glutathione S-transferase (*GSTO1*), microsomal glutathione S-transferase 1 (*MGST1*), and nuclear factor erythroid 2-related factor 2 (*NFE2L2*), all proteins that promote responses to oxidative stress [47], suggesting a potential for reduced oxidative stress defenses during cold acclimation and freezing. Furthermore, glutathione-specific gamma-glutamylcyclotransferase 1 (*CHAC1*), which has been shown to increase oxidative stress by degrading glutathione, was upregulated in cold and frozen frogs. Several organisms upregulate antioxidant responses seasonally or in response to cold exposure [48, 49]. Antioxidant enzyme levels have not been investigated in *D. chrysoscelis* but may be constitutively elevated as reported in other freeze-tolerant frogs [8].

Cold exposure and freezing also modulated the hepatic transcriptome of *D. chrysoscelis* by promoting anti-apoptotic and DNA-repair responses. Caspase 3 (*CASP3*) which is involved in apoptosis [50], was downregulated in cold and frozen frogs, whereas the dual specificity protein phosphatase 10 gene (*DUSP10*), which inhibits p38 MAP kinase and contributes to regulation of apoptosis [51], was also upregulated in both cold and frozen animals. *GADD45*, a protein that promotes growth arrest and DNA repair, is responsive to hypoxia [52], hyperosmotic stress [9], and is also involved in apoptosis [52], was upregulated in cold and frozen frogs.

### Differential expression of genes related to nucleotide and nucleic acid metabolism

Regulators of transcription and translation were responsive to cold and freezing. Transcription factors can influence transcription by interacting with other transcription factors and RNA polymerase, and by changing the structure of chromatin [53]. The downregulation of several transcription factors, including *SOX30* and *NFE2L2*, suggests a possible mechanism for transcriptional repression. Furthermore, downregulation of the transcription activator *STAT2* [54], and *EEF1A1*, a key participant in translation and regulator of proteolysis, indicates other potential mechanisms for regulating transcription and translation beyond changes in transcription factors. Decreased levels of *EEF1A1* protein have also been observed in cold-exposed *X. laevis* [55] and in winter-acclimatized *Rana sylvatica* [10].

### Differential expression of genes involved in protein, amino acid, and lipid metabolism

Levels of several transcripts involved in the ubiquitin proteasome pathway, including *PJA2, RNFL30*, *UBE2D1*, and *RBBP6* were upregulated in cold and frozen frogs, whereas *FBX15* expression also increased from the cold to the frozen states. The conjugation of ubiquitin with target proteins promotes binding to the proteasome and subsequent protein degradation. Protein ubiquitination is transcriptionally enhanced in cold-exposed fish [48, 56, 57], and frogs [55], possibly due to cold-induced denaturation [57], and may serve as a marker for cold exposure [48]. Protein turnover in cold *D. chrysoscelis* could supply the cell with amino acids to be used for synthesis of ATP, carbohydrates, and proteins, or these might serve as cryoprotectants [2] or as sources of cryoprotective urea [15]. Given the low oxygen availability in frozen frogs [3], ATP synthesis from sources other than carbohydrates is unlikely in this state. Moreover, transcripts involved in amino acid trafficking (e.g. *GPT2* and *GLUD1*), and carbohydrate flux (*GAPDH*) were downregulated in cold and frozen animals, suggesting amino acids derived from proteolysis may be accumulating in the liver, warranting further investigation of hepatic protein metabolism during this period.

Several transcripts associated with β-oxidation of fatty acids were downregulated in our cold and frozen animals, including *ACAD10*, *EHHADH*, and *DECR1*, as well as transcripts of *DGAT1*, which is essential for triglyceride synthesis. Downregulation of these transcripts may be related to the overall metabolic depression occurring in cold animals, as it curtails unnecessary energetic expenditures incurred by transcription [58]. Furthermore, the predominance of anaerobic metabolism during freezing precludes lipid use for ATP synthesis [3]. Surprisingly, expression of *ACACB*, involved in fat storage, and *DGAT2* increased in cold and frozen frogs.

### Differential expression of non-coding RNAs

Approximately 3.6% of the differentially expressed sequences identified in this study were non-coding and showed no homology to sequences in existing databases. In the absence of a reference genome for sequence alignment, it is not possible to determine how many unique transcripts are represented by the 5800 sequences that exhibited differential regulation. The effect of cold exposure and freezing on levels of non-coding RNAs has been investigated in multiple taxa, with particular focus on certain miRNAs [38, 39]. Cold exposure and freezing increased the expression of miR-30 and miR-142 in *D. chrysoscelis*. In frozen *R. sylvatica,* miR-30 levels increased with freezing in skeletal muscle [38]; this microRNA has several demonstrated effects, including inhibiting apoptosis [59] and protecting against liver fibrosis [60], but its role in freeze tolerance remains to be elucidated. In contrast, most miRNAs that were differentially regulated in the brain of *R. sylvatica* had reduced expression in frozen animals, which may suggest a neuroprotective role for those molecules [39].

Several snoRNAs were responsive to freezing (Table 6). SnoRNAs may have important roles in regulating transcription and translation by modifying rRNA, and by functioning like miRNA [61], but their role in cold acclimation and freezing is unknown. SnoRA5, which was upregulated in frozen frogs, is associated with kidney reperfusion injury [62], and its upregulation in frozen animals may be related to the cessation of circulation that occurs in that condition [2].

In addition to the identified non-coding RNAs, it is likely that among the transcripts in this subset are long non-coding RNAs (lncRNAs) that may contribute to the process of freeze tolerance through mechanisms that remain to be defined. LncRNAs are broadly characterized as non-conserved, transcribed RNAs > 200 bp in length that can reside in the nucleus or in the cytoplasm and can interact with chromatin as well as ribosomes. LncRNAs regulate gene expression through a diversity of mechanisms, including gene silencing, epigenetics, transcriptional and post-transcriptional regulation, RNA processing and transport (reviewed in [63]). Moreover, tissue and condition specific differential expression of lncRNAs has been documented in a number of biological and pathophysiological conditions, indicating that regulating the expression of lncRNAs is important to function [64]. Sequence analysis of the North American bullfrog genome identified 6223 putative lncRNAs, a subset of which exhibited thyroid responsive DGE, indicating that lncRNAs are present across the anuran order and are subject to differential regulation [65]. LncRNAs have been implicated in cold and freeze response mechanisms in both plant and animal systems. Cold-dependent differential expression and differential alternative splicing of lncRNAs have been shown to correlate with acclimation and freeze tolerance in *Arabidopsis* [66, 67]. Moreover, differential expression of lncRNA transcripts and associated target genes involved in cold response and apoptosis in frozen and thawed giant panda (*Ailuropoda melanoleuca*) sperm exhibited co-expression regulatory patterns, suggesting a role for the interaction between lncRNAs and their target mRNAs in cryopreservation, and as an extension, freeze tolerance [68]. Experiments designed to determine the cis- and trans-targets of these putative lncRNAs, and to characterize the potential differentially expressed genes and co-regulation patterns of lncRNAs and target transcripts involved, are planned.

### Conservation and specialization of transcriptomic regulation in the cold

Transcriptomic analyses have been used to identify gene regulatory pathways involved in cold and freeze tolerance in other organisms, including hibernating mammals, plants, fishes, insects and other invertebrates. From these studies, diverse functional pathways and processes subject to cold-responsive control have been identified. Hibernation, characterized by alternating periods of euthermia and hypothermia in endotherms, appears to have a complex of both common and species-specific responses. For example, transcriptomic studies in skeletal muscle, adipose tissue and liver from hibernating grizzly bears (*Ursus arctos horribilis*) revealed differential expression for genes involved in aspects of metabolism (decrease in insulin responsive pathways, upregulation of muscle anabolic pathways), as well as changes in the expression of lncRNAs, in addition to tissue-specific regulatory responses [69]. Thirteen-lined ground squirrels (*Ictidomys tridecemlineatus*) also differentially express genes in skeletal muscle during hibernation [70]; however, the particular pathways that are regulated (e.g., protein turnover pathways) differ from those in grizzly bear. Likewise, during discrete phases of their hibernation cycle, white adipose tissue from dwarf lemurs (*Cheirogaleus medius*) differentially expressed genes related to metabolism, feeding, circadian rhythms and coagulation [71], a suite of responses that differs from that in adipose tissue of grizzly bears. These findings suggest hibernators may have evolved both common as well as tissue- and species-specific strategies for survival, perhaps related to aspects of body size, metabolic strategy, and natural and phylogenetic history.

In ectotherms, too, transcriptional regulation during cold conditions includes both responses that are common across animals and those that are tissue- or species specific. In threespine stickleback (*Gasterosteus aculeatus*)*,* cold acclimation resulted in an upregulation in skeletal muscle genes involved in RNA and protein processing and cell cycle regulation and downregulation of transcripts encoding extracellular matrix, angiogenic, and contractile proteins [72]. Analyses of gill and liver transcriptomes from cold-tolerant and cold-sensitive blue tilapia (*Oreochromis aureus*) showed similar patterns of down regulation of cell-matrix interactions and upregulation of proteolytic enzymes, as well as opposing patterns of upregulation of glycolysis/gluconeogenesis in the gill and amino acid biosynthesis in the liver of cold-sensitive fish, and downregulation of these processes in cold-tolerant fish [73]. More diversity of response is apparent if one extends the comparison to invertebrates (e.g., *Drosophila melanogaster*, in which cold acclimation induced differential regulation in one third of the transcriptome and one half of the metabolome [74]) or plants (in which pathways for biosynthesis of secondary metabolites, hormone signal transduction, plant-pathogen interaction, and stress responses all are involved in cold-tolerance [75–77]).

In the treefrogs we studied, we identified few differences in gene expression between cold and frozen animals. Nevertheless, there may be categorically important differences between these states in other circumstances or organisms. For example, freeze tolerance in the terrestrial worm (*Enchytraeus albidus*)*,* is characterized by differential expression of genes involved in anion transport, metabolism, oxidative and metabolic stress reduction, cryoprotectant solute transport and signal transduction [78]. Similarly, differentially expressed genes involved in freeze tolerance of the cricket *Gryllus veletis* are associated with metabolism, cryoprotectant solute transport, cytoskeleton stability, oxidative and metabolic stress reduction, signal transduction, and cellular processes [79]. It is apparent that the detailed responses to cold and freezing differ among organisms, or even among strains of organisms, and also reflect specialized or condition-specific gene regulatory cascades that are unique and specialized [78, 80, 81]. As described above, transcripts for several genes related to heat shock protein response, DNA repair, and the ubiquitin proteasome pathway showed an increase in abundance in cold and frozen frogs, whereas transcripts for genes involved in oxidative stress and anoxia response, both potential sources of cellular damage during freezing, showed either a reduced abundance or remained unchanged. These transcriptomic results are consistent with correlative trends identified in hepatoproteome studies of thermal responses in *R. sylvatica* [10]. Interestingly, similar proteomic responses were observed for a subset of genes related to glycogen synthesis and carbohydrate metabolism in response to low temperature exposure (5 °C) in the non-freeze tolerant anuran *Xenopus laevis* [55]. Beyond transcriptional control, it is also clear from other studies in *R. sylvatica* that thermal condition dependent regulation of protein expression is influenced by tissue-specific regulation and post-translational modification [82, 83]. Further studies will be required to more broadly define the relation between transcriptomic regulation and protein expression as animals freeze and thaw.

## Conclusions

*Dryophytes chrysoscelis* survives winter’s cold by tolerating freezing of its body fluids, a strategy that requires both a preparatory cold acclimation and a series of cryoprotective responses triggered upon ice nucleation. Hepatic cryoprotectant production during cold acclimation and freezing is of paramount importance to ensure freezing survival. This was the first study to employ RNA-Seq to analyze the freezing response in a freeze-tolerant vertebrate. This study demonstrates that the hepatic transcriptome of *Dryophytes chrysoscelis* is responsive to cold and freezing with differential expression of numerous genes that contribute to cryoprotectant mobilization, stress response, and transcriptional regulation. The multiple physiological challenges associated with freezing exposure, as well as the transcriptomic responses observed in this study, are shared with several organisms that face similar ecophysiological circumstances [3, 23, 25, 44], suggesting a common regulatory framework. The extent to which these changes in mRNA expression are reflected at the level of the proteome, and the contributions of those changes relative to other cellular responses, remain to be defined. Intriguingly, this study also identified numerous non-coding RNAs that were differentially expressed in response to cold and freezing; the roles of those molecules deserve further study.

## Methods

### Experimental animals

Twelve male *D. chrysoscelis* were collected from ponds in southwest Ohio, USA, within a ten mile radius of Caesar Creek State Park Wildlife Area (39.5065512,-84.0604201) during summer months (May–July). Wild caught frogs (age unknown, weight ~ 7 g) were collected under a permit from the Ohio Division of Wildlife. Frogs were transported to the laboratory at Wright State University and were kept in plastic containers with free access to water. Initially, frogs were warm-acclimated to 22 °C under a 12:12 h light-dark regime, fed crickets thrice weekly, and provided water *ad libitum*. A random group of these frogs (*n* = 4) was sampled after a minimum of 8 weeks in acclimation (herein called “warm frogs”). In fall, a random subset of animals was transferred to a cold room and cold-acclimated by being progressively cooled to 5 °C, with an accompanying shift to a 8:16 h light-dark regime, over a 2-month period as previously described [5]. During cold acclimation, each frog was fed until it no longer accepted food, which occurred once the acclimation temperature was 8 °C and lower. In winter, a random subset of the cold-acclimated frogs was sampled (*n* = 4) (hereafter “cold frogs”) while another subset was used in freezing experiments. If a frog exhibited characteristics outside the normal observed characteristics, the frog was removed from the study in consultation with a laboratory animal research veterinarian and given treatment. Characteristics that would be cause for removal include atypical body posturing, outstretched legs, poor response to stimulus (depending on acclimation state) or other atypical physical characteristics that could be considered symptomatic of disease. Animals to be frozen were placed in individual plastic containers in an incubator set at 2 °C. The temperature of the incubator was then lowered to − 2 °C at a rate of 1 °C day^− 1^. Once the incubator reached − 2 °C, the unfrozen frogs were individually placed on top of moist gauze and ice was added to the individual containers to ensure crystallization. Immediately after ice was added to the containers, the incubator temperature was lowered to − 2.5 °C. Frogs were visually inspected 24 h post-inoculation for signs of internal ice formation (stiff body and limbs, presence of subcutaneous ice crystals noticeable in the ventral and dorsal surfaces) and sampled shortly thereafter (n = 4) (“frozen frogs”). Internal ice formation was confirmed during tissue sampling by the presence of large pieces of ice surrounding digestive organs, and sheets of ice between the skin and skeletal muscles.

### Liver sampling and total RNA isolation

Animals were euthanized following approved WSU Laboratory Animal Care and Use Committee (LACUC) protocols. Warm-acclimated animals were anesthetized by being placed in a shallow volume of skin permeable 0.1% Tricaine-S (MS-222; Pentair Aquatic Systems, Cary NC) in phosphate buffered saline, a well-documented and routinely used anesthetic for amphibians. The anesthetic was taken up by the frog through the pelvic patch. Animals were monitored as they became lethargic and lowered their body further. They were then decapitated before pithing the spinal column. Cold-acclimated animals and frozen animals were directly decapitated and pithed. Warm-acclimated animals were euthanized and dissected at room temperature (22 °C) in an LACUC-approved laboratory space. Cold-acclimated animals and frozen animals were euthanized and dissected in a 4 °C cold room. Animals were euthanized between 10:00 am and 4:00 pm, local EST. The liver was excised and immediately frozen in liquid N_2_. Samples were stored at − 80 °C until RNA isolation. Total RNA was isolated from 10 to 20 mg of liver using the Qiagen RNAeasy Mini Kit (Qiagen, Hilden, Germany). Tissue samples were homogenized with a hand-held homogenizer with a rotating blade, in isolation buffer, and RNA was isolated following the manufacturer’s instructions. Extracted RNA was treated with DNase I (Ambion, Thermo Fisher Scientific, Waltham, MA, USA) to eliminate potential genomic DNA contamination and precipitated using lithium chloride (Sigma, St. Louis, MO, USA). RNA concentration and quality were determined with a nanophotometer (Denville Scientific Inc., Holliston, MA, USA). Total RNA from 12 male frogs (*n* = 4 per treatment) was submitted to Cofactor Genomics (http://cofactorgenomics.com/, St. Louis, MO) to be used in high-throughput RNA-Sequencing. Sample size determination was based on statistical analyses of prior gene expression results [7]. As the frogs were wild caught and their ages and degree of inter-relatedness were unknown, it was anticipated that inter-individual variability, independent of acclimation state, but related to age, acute physiological condition, and/or genetic variability, would be observed. Thus, a sample size that slightly exceeds the power analysis generated from prior results was used in this experimental design to address this predicted inter-individual variation.

### mRNA purification and next generation sequencing library preparation

Preparation of mRNA library and Next Generation Sequencing were performed by Cofactor Genomics. Samples used for library preparation had an RNA integrity number (RIN) > 7, a UV absorption ratio A_260_/A_280_ > 1.9, and A_260_/A_230_ > 2. Total RNA samples were incubated with mRNA capture beads to remove contaminating ribosomal RNA using the Kapa Stranded mRNA-Seq kit (Kapa Biosystems, Roche, Pleasanton, CA) following manufacturer’s instructions. The resulting poly(A)-captured mRNA was then fragmented. First-strand cDNA synthesis was performed using random primers and reverse transcriptase in the presence of Actinomycin D, followed by second-strand cDNA synthesis with Rnase H and DNA polymerase I. Double-stranded cDNA was end-repaired and A-tailed for subsequent adaptor ligation. Indexed adaptors were ligated to the A-tailed cDNA. Enrichment by PCR was performed to generate the final cDNA sequencing library. Libraries were sequenced as single-end 75 base pair reads on an Illumina NextSeq500 following the manufacturer’s protocols.

### Transcriptome assembly and annotation

Transcriptome assembly and annotation were performed by Cofactor Genomics. Raw sequence data (in FASTQ format) were assessed for quality using FastQC and ribosomal RNA content (sortmeRNA, http://bioinfo.lifl.fr/RNA/sortmerna/). A transcriptome reference was created through the de novo assembly of adapter- and quality-filtered reads. As a foundation for the assembly, Cofactor’s pipeline used the Trinity Suite (Broad Institute, version r20140717) with optimized parameters, resulting in transcript models for alternatively spliced isoforms [84]. For each transcript or patch, the number of mapped reads was determined using Kallisto [85] and used for statistical analysis.

The de novo assembly was annotated by searching the Uniprot Swiss-Prot database via BLASTX (or TBLASTX when appropriate), following the Trinotate pipeline, with an expectation value (e-value) of 1 × 10^− 5^ [31]. The transcriptome was also annotated using Blast2GO (BioBam, Spain). Transcripts were imported into Blast2GO and NCBI BLASTX was used to search each transcript against the NCBI vertebrate, non-redundant (nr) protein database with an e-value of 1 × 10^− 3^, and against the reference protein sequences (RefSeq) for *Xenopus sp*. The highest scoring BLAST hit was used to assign a gene ID to each transcript. The number of genes identified was estimated by determining the number of distinct gene IDs (Uniprot IDs and NCBI accession numbers) in the transcriptome; transcripts assigned to distinct identifiers were considered different genes. For transcripts with a BLAST match, GO terms were retrieved using the GO-mapping tool (e-value of 1 × 10^− 6^) and InterProScan tool in Blast2GO, and the annotations from these two tools were merged. To ensure maximal transcriptome coverage, transcriptome annotation performed with Trinotate was merged with the annotation performed in Blast2GO. The remaining unidentified transcripts were searched in the Rfam database, which contains a collection of RNA sequence families of structural RNAs such as non-coding RNA genes, using Blast2GO.

### Differential expression analysis

The abundance of annotated transcripts generated in Kallisto were uploaded to R using the package *tximport* [40]. Differential gene expression was determined at the gene level, and at the transcript level by selecting gene-level summary of the Kallisto data [40]. Differentially expressed genes were also identified from transcript-level data by evaluating if at least one transcript isoform of a gene was differentially expressed, followed by aggregation of transcripts at the gene level [40]. Differential expression among samples was determined using the DESeq2 [34] package for R [86] in Rstudio (v. 1.0.136). Data were normalized with DESeq2 algorithms. Transcripts with less than one raw count were excluded, and the default DESeq2 algorithms were used to remove outlier transcripts based on Cook’s distances [87]. The Benjamini and Hochberg algorithm [88] was used to control the false discovery rate (FDR) and transcripts were considered differentially expressed if the expression between two treatments differed by at least |2|-fold with FDR < 0.05.

Differential transcript expression was also determined for the entire transcriptome (including non-annotated transcripts) to determine if non-coding RNAs showed differential expression. The abundance of all transcripts generated in Kallisto was uploaded to R using the package *tximport* and the analysis was performed as detailed for annotated transcripts, except differential gene expression was only determined at the transcript level without gene-level summarization. We then searched the differentially expressed transcripts for non-coding RNAs identified in the Rfam database.

### Functional classification and gene ontology (GO) enrichment analysis

The Protein Analysis Through Evolutionary Relationships (PANTHER) Classification System [89] was used to functionally classify the annotated transcriptome. Classification was performed to determine GO slim categories for Biological Process (BP), Molecular Function (MF), Cellular Component (CC). Slim categories were chosen as opposed to traditional GO categories to provide summarized overview of the transcriptome.

Genes that were differentially expressed based both on the gene and transcript-level approaches were analyzed in PANTHER to determine their GO slim BP categories. GO enrichment analysis was also performed on these differentially expressed genes using the Database for Annotation, Visualization and Integrated Discovery (DAVID v6.8) [90, 91]. The annotated transcriptome was used as background. Significant enrichment of GO Biological Process categories was tested and categories were considered enriched if the FDR < 0.05.

## Supplementary information

**Additional file 1: Table S1.** genes differentially regulated in *Dryophytes chrysoscelis* compared to warm animals with corresponding gene symbol, UniprotID, Transcript ID, and fold-change in each treatment. **Table S2.** genes differentially expressed in frozen *Dryophytes chrysoscelis*, relative to cold animals, with corresponding gene symbol, UniprotID, Transcript ID, and fold-change.

## Data Availability

The RNA-Seq data generated and analyzed in this study are available in the National Center for Biotechnology Information (NCBI) Sequence Read Archive (SRA), BioProject ID number PRJNA603387, (https://www.ncbi.nlm.nih.gov/sra/?term=PRJNA603387), BioSample accession numbers SAMN13930926-SAMN13930949, Experiment accession numbers SRX7650130-SRX7650153, Run accession numbers SRR10988709-SRR10988686.

## Abbreviations

BLAST: Basic local alignment search tool
BP: Biological process
DAVID: Database for annotation, visualization and integrated discovery
GO: Gene ontology
lncRNAs: long non-coding RNAs
miRNA: micro RNAs
PANTHER: Protein analysis through evolutionary relationships
RNA-seq: High throughput RNA sequencing
SNORD: Small nucleolar RNAs, C/D Box
