# Supplementary material for: Hepatic transcriptome of the freeze-tolerant Cope’s gray treefrog, Dryophytes chrysoscelis: responses to cold acclimation and freezing
Source: BMC Genomics. 2020 Mar 12;21:226. doi: 10.1186/s12864-020-6602-4 (PMC7069055; doi:10.1186/s12864-020-6602-4)
Supplement: Supplementary file 1 — Additional file 1: Table S1. genes differentially regulated in Dryophytes chrysoscelis compared to warm animals with corresponding gene symbol, UniprotID, Transcript ID, and fold-change in each treatment. Table S2. genes differentially expressed in frozen Dryophytes chrysoscelis, relative to cold animals, with corresponding gene symbol, UniprotID, Transcript ID, and fold-change. [file 12864_2020_6602_MOESM1_ESM.docx]

Table S1. Genes differentially regulated in *Dryophytes chrysoscelis*

| Category | Gene symbol | Gene name | UniProt ID | Transcripts | Fold-change in Cold | Fold-change in Frozen |
| --- | --- | --- | --- | --- | --- | --- |
| Carbohydrate metabolism |  |  |  |  |  |  |
|  | *GAPDH* | Glyceraldehyde-3-phosphate dehydrogenase | G3P_HUMAN  G3P_PONAB | c114101_g1_i1  c114101_g2_i1 | -4.1  -5.0 | -4.4  -4.8 |
|  | *G6PC* | Glucose-6-phosphatase | G6PC_HAPNU | c76622_g1_i2 | - | 4.1 |
|  | *GCKR* | Glucokinase regulatory protein | GCKR_XENLA | c103023_g1_i1  c103023_g1_i3 | -4.8  - | -4.8  -5.3 |
|  | *GLPK* | Glycerol kinase | GLPK_HUMAN | c140868_g1_i1 | -6.2 | -6.5 |
|  | *GK5* | Putative glycerol kinase 5 | GLPK5_XENLA | c114472_g1_i3 | 5.7 | 4.8 |
|  | *HK2* | Hexokinase-2 | HXK2_MOUSE | c113474_g2_i4 | 6.5 | 6.4 |
|  | *PDK2* | Pyruvate dehydrogenase (acetyl-transferring)] kinase isozyme 2, mitochondrial | PDK2_RAT | c102885_g1_i2 | 5.5 | 6.0 |
|  | Phka2 | Phosphorylase b kinase regulatory subunit alpha, liver isoform | KPB2_MOUSE | c115024_g1_i3 | - | 5.8 |
|  | *SLC2A8* | Solute carrier family 2, facilitated glucose transporter member 8 | GTR8_RAT | c102491_g1_i3 | - | -2.9 |
|  | *SLC3A10* | Solute carrier family 2, facilitated glucose transporter member 10 | GTR10_XENTR | c99168_g1_i2 | -3.2 | -3.1 |
| Transport | *SLC5A9* | Sodium/glucose cotransporter 4 | SC5A9_MOUSE | c91511_g1_i1  c131517_g1_i1 | -  - | -4.8  -4.5 |
|  | *PHKG2* | Phosphorylase b kinase gamma catalytic chain, liver/testis isoform | PHKG2_HUMAN | c101305_g1_i1  c88287_g1_i1 | -3.0  -2.8 | -3.6  -3.2 |
|  | *PFKM* | ATP-dependent 6-phosphofructokinase, muscle type | PFKAM_HUMAN | c108491_g1_i3 | -2.3 | - |
| Stress |  |  |  |  |  |  |
|  | *CAT* | Catalase | CATA_GLARU | c111503_g2_i1  c111503_g1_i4  c92630_g1_i1 | -3.7  -  -3.5 | -4.9  -3.5  -4.5 |
|  | *DUSP10* | Dual specificity protein phosphatase 10 | DUS10_BOVIN | c103298_g1_i1  c115638_g1_i1 | 3.3  4.0 | -  3.8 |
|  | *GADD45* | Growth arrest and DNA damage-inducible protein alpha | GA45A_FELCA | c76805_g1_i1  c84137_g1_i2  c85034_g1_i1 | 4.0  7.4  2.5 | 2.7  6.0  - |
|  | *HSP90AA1* | Heat shock protein HSP 90-alpha | HS90A_BOVIN | c99478_g1_i1 | 3.1 | - |
|  | *HSP70* | Heat shock 70 kDa protein | HSP70_XENLA  HSP70_PLEWA | c109798_g2_i2  c114734_g1_i1  c85408_g1_i1  c109798_g2_i4  c91406_g1_i1  c87025_g1_i1 | 5.2  5.6  6.1  6.3  8.0  6.3 | -  -  -  -  -  - |
| Cellular process |  |  |  |  |  |  |
|  | *CASP3* | Caspase 3 | CASP3_BOVIN | c105093_g2_i1 | -6.4 | -7.5 |
|  | *GSTO1* | Glutathione S-transferase omega-1 | GSTO1_PIG | c112015_g4_i8 | - | -3.4 |
|  | *HSPA5* | 78 kDa glucose-regulated protein | GRP78_CHICK | c105829_g2_i3 | 5.7 | 4.8 |
|  | *MGST1* | Microsomal glutathione S-transferase 1 | MGST1_PIG | c97325_g1_i1 | -3.1 | -3.4 |
|  | *PRKACB* | β catalytic subunit of cAMP-dependent protein kinase | KAPCB_HUMAN | c102658_g2_i1 | - | 2.3 |
| Nucleotide and nucleic acid metabolism |  |  |  |  |  |  |
|  | *EEF1A1* | Elongation factor 1-alpha 1 | EF1A1_RABIT | c811_g1_i1 | -4.7 | -6.1 |
|  | *NFE2L2* | Nuclear factor erythroid 2-related factor 2 | NF2L2_HUMAN | c44920_g1_i1 | - | -2.3 |
|  | *RBBP6* | E3 ubiquitin-protein ligase RBBP6 | RBBP6_HUMAN | c113216_g2_i3 | 2.6 | 2.6 |
|  | *SOX30* | Transcription factor SOX-30 | SOX30_HUMAN | c116368_g1_i1 | - | -3.2 |
|  | *STAT2* | Signal transducer and activator of transcription 2 | STAT2_HUMAN | c114648_g1_i3  c114648_g1_i5 | -4.2  -4.8 | -4.4  -6.3 |
| Protein metabolism |  |  |  |  |  |  |
|  | *HSPH1* | Heat shock protein 105 kDa | HS105_HUMAN | c110306_g1_i1  c110306_g1_i2 | 4.6  2.7 | -  3 |
|  | *PJA2* | E3 ubiquitin-protein ligase Praja-2 | PJA2_MOUSE | c110455_g1_i8 | - | 6.7 |
|  | *UBE2D1* | Ubiquitin-conjugating enzyme E2  D1 | UB2D1_RAT | c112378_g3_i1 | - | 6.4 |
|  | Rnf130 | E3 ubiquitin-protein ligase RNF130 | GOLI_MOUSE | c109788_g1_i1 | 7.0 | 7.5 |
| Amino acid metabolism |  |  |  |  |  |  |
|  | *GLUD1* | Glutamate dehydrogenase 1, mitochondrial | DHE3_HUMAN | c50160_g1_i1 | -3.8 | -3.6 |
|  | *CHAC1* | Glutathione-specific gamma-glutamylcyclotransferase 1 | CHAC1_HUMAN CHAC1_MOUSE | c108449_g1_i3  c108449_g1_i2 | 4.2  4.5 | 3.8  - |
|  | *GPT2* | Alanine aminotransferase 2 | ALAT2_XENTR  ALAT2_XENLA | c83952_g1_i1  c98215_g1_i2  c34937_g1_i1  c66918_g1_i1 | -6.1  -3.8  -2.6  - | -7.9  -3.8  -2.9  -3.8 |
| Lipid metabolism |  |  |  |  |  |  |
|  | *ACACB* | Acetyl-CoA carboxylase | ACACB_MOUSE | c113900_g2_i2 | 9.0 | 9.6 |
|  | *ACAD10* | Acyl-CoA dehydrogenase family member 10 | ACD10_HUMAN | c111672_g1_i1 | -3.4 | - |
|  | *GPAT4* | Glycerol-3-phosphate acyltransferase 4 | GPAT4_MOUSE  GPAT4_PONAB | c10257_g1_i1  c101384_g1_i1 | 3.6  2.1 | 3.1  2.5 |
|  | *DECR1* | 2,4-dienoyl-CoA reductase, mitochondrial | DECR_HUMAN | c98556_g1_i1 | -3.2 | -3.6 |
|  | *DGAT1* | Diacylglycerol O-acyltransferase 1 | DGAT1_BOVIN | c110916_g1_i2 | - | -4.5 |
|  | *DGAT2* | Diacylglycerol O-acyltransferase 2 | DGAT2_XENTR | c107722_g1_i1 | - | 2.8 |
|  | *EHHADH* | Peroxisomal bifunctional enzyme | ECHP_DANRE | c101886_g1_i4 | -3.7 | - |

Genes differentially (in both the transcript-level and gene-level analyses) expressed relative to the warm condition (log2-fold change ≥|2|, FDR<0.05). If log2 fold-change <0, genes were downregulated relative to warm controls; if log2 fold change >0, genes were upregulated relative to warm controls. Blank entries result from absence of differential expression at the gene or transcript-levels analysis, or both.

Table S2. Genes differentially expressed in frozen *Dryophytes chrysoscelis*, relative to cold animals

| Category | Gene symbol | Gene name | UniProt ID | Transcripts | Fold-change in Frozen |
| --- | --- | --- | --- | --- | --- |
|  | C1QBP | Complement component 1 Q subcomponent-binding protein, mitochondrial | C1QBP_BOVIN | c43907_g1_i1 | 6.1 |
|  | fbxl5 | F-box/LRR-repeat protein 5 | FBXL5_XENTR | c34163_g1_i1 | 7.9 |
|  | N/A | Veficolin-1 | FCNV1_VARKO | c110368_g1_i15 | 6.7 |

Genes differentially expressed relative to the cold condition (FDR<0.05). If log2 fold-change <0, genes were downregulated relative to cold condition; if log2 fold change >0, genes were upregulated relative to cold condition. Genes with parenthetic categories were not depicted in figure 5.
